# Host Resistance to Parasitic Plants—Current Knowledge and Future Perspectives

**DOI:** 10.3390/plants12071447

**Published:** 2023-03-25

**Authors:** Ivanela A. Albanova, Lyuben I. Zagorchev, Denitsa R. Teofanova, Mariela K. Odjakova, Lyudmila I. Kutueva, Vasily V. Ashapkin

**Affiliations:** 1 Faculty of Biology, Sofia University “St. Kliment Ohridski”, 8 Dragan Tsankov Blvd., 1164 Sofia, Bulgaria; 2 Belozersky Institute of Physico-Chemical Biology, Lomonosov Moscow State University, Moscow 119234, Russia

**Keywords:** *Cuscuta*, epigenetics, host resistance, parasitic plants, *Striga*, transgenerational acquired resistance

## Abstract

Parasitic flowering plants represent a diverse group of angiosperms, ranging from exotic species with limited distribution to prominent weeds, causing significant yield losses in agricultural crops. The major damage caused by them is related to the extraction of water and nutrients from the host, thus decreasing vegetative growth, flowering, and seed production. Members of the root parasites of the Orobanchaceae family and stem parasites of the genus *Cuscuta* are among the most aggressive and damaging weeds, affecting both monocotyledonous and dicotyledonous crops worldwide. Their control and eradication are hampered by the extreme seed longevity and persistence in soil, as well as their taxonomic position, which makes it difficult to apply selective herbicides not damaging to the hosts. The selection of resistant cultivars is among the most promising approaches to deal with this matter, although still not widely employed due to limited knowledge of the molecular mechanisms of host resistance and inheritance. The current review aims to summarize the available information on host resistance with a focus on agriculturally important parasitic plants and to outline the future perspectives of resistant crop cultivar selection to battle the global threat of parasitic plants.

## 1. Parasitic Plants and Agriculture

Parasitic angiosperms represent a broad group of more than 4500 species, distributed into 12 families and approximately 300 genera [1]. Regardless of the degree of parasitism, e.g., full or partial dependence on the host plant, they all share a common feature called the haustoria, the functional physiological link between hosts and parasites [2,3]. Through it, they acquire both mineral nutrients and photosynthates from their hosts, but also bi-directionally exchange signaling molecules, genetic material, and also pathogens [4].

Although overall harmful, not all parasitic plants are equally damaging to their hosts from the perspective of human activity. Most of them have a limited distribution and limited impact and are regarded as exotic, or even endangered species [5,6]. Parasitic plants are also widely employed in traditional medicine [7,8], and largely shape and contribute to the diversity in natural plant communities [7,9]. Interestingly, some can be cultivated on purpose, either as a food source [10], for medicinal purposes [11], or as a bio-restoration tool to increase species diversity [12].

On the other hand, relatively few species of parasitic plants represent some of the most damaging agricultural pests, causing USD billions of annual losses, food insecurity, and ecological threats. They largely belong to the Convolvulaceae family, genus *Cuscuta*, and the Orobanchaceae family, genera *Striga* and *Orobanche*. *Cuscuta* spp., or dodders, are approximately 200 species of stem holoparasites (e.g., non-photosynthesizing) with worldwide distribution [13]. Relatively few of them, however, are considered damaging to agricultural crops [14], among which *Cuscuta campestris* Yunck. is the most prominent, with a worldwide distribution. They are reported in numerous crop plants, mostly dicotyledons such as sugar beet, alfalfa, tomato, etc. [15]. Other economically important members of the genus include *C. reflexa* Roxb., *C. monogyna* Vahl, *C. gronovii* Willd. ex Roem. et Schult., *C. chinensis* Lam., and *C. epithymum* L. Due to their generalist nature of parasitism, i.e., infecting a wide range of host plants, the impact of *Cuscuta* spp. may be enormous and unpredictable. For example, *C. reflexa* is an emerging threat to tea, coffee, and mango production in Eastern Africa [16], *C. campestris* is a well-known pest on sugar beet in many European countries [17], and *C. gronovii* is damaging cranberry production in North America [18]. Yield losses may reach 50–70% of the expected [19,20], and the quality of the yield may be also negatively affected [21].

Family Orobanchaceae consists of over 2000 species in more than 100 genera, ranging from non-photosynthetic root holoparasites and photosynthetic root hemiparasites to the non-parasitic *Lindenbergia* and *Rehmannia-Triaenophora* clades [22]. The most damaging are members of the *Striga*, *Orobanche*, and *Phelipanche* genera [15]. Witchweeds (*Striga* spp.) are several species of root hemiparasites, among which *Striga asiatica* (L.) Kuntze and *Striga hermonthica* (Delile) Benth. pose the greatest threat, especially in Africa and Asia, where they are severely affecting cereal production. Crop loss may reach 80% yield reduction to complete loss in various staple crops, such as maize, rice, sorghum, and millet, thus representing one of the major food security threats, especially in Africa. Among the root holoparasitic broomrapes (*Orobanche* and *Phelipanche* spp.), *O. crenata* Forssk., *O. ramosa* L., and *O. cernua* Loefl., among others, are prominent parasites on various crop plants of the Apiaceae, Fabaceae, and Solanaceae families, causing yield losses of between 5 and 100% [23]. A special case within the broomrapes is *O. cumana* Wallr., an exclusive parasite on sunflower and a significant agricultural pest in Europe [24], which can also lead to a complete loss of yield. The most damaging parasitic plants are further listed in Table 1.

**Table 1:** List of the most damaging parasitic plants and their main affected crop plants.

Parasitic Plant Family	Common Name	Latin Name	Most Affected Crop Plants	References
Convolvulaceae	Dodder	*Cuscuta campestris*	Legumes (Fabaceae) Sugarbeet (*Beta vulgaris*)	[17,25,26] [17,21]
Orobanchaceae	Sunflower broomrape Broomrapes Witchweeds	*Orobanche cumana* *Orobanche*/*Phelipanche* spp. *Striga asiatica*/*gesneroides*/*hermonthica*	Sunflower (*Helianthus annuus*) Tomato (*Solanum lycopersicum*) Pea (*Pisum sativum*) Maize (*Zea mays*) Sorghum (*Sorghum bicolor*) Rice (*Oryza sativa*)	[24] [27] [28] [29] [30] [31]

Apart from the well-established parasitic pests, there are also several parasites with apparent restricted influence, but which are also significant to agriculture. *Alectra vogelii* (Benth.), of the family Orobanchaceae, is affecting mainly legumes in Africa and may cause between 50 and 100% yield loss in crops [32]. Another relative, *Rhamphicarpa fistulosa* (Hochst.) Benth., is establishing itself as an increasing problem for rice production [33], causing up to 60% yield loss and annual economic losses of USD 175 million. A relatively recent report claimed that *Cassytha filiformis* L. (Lauraceae), convergently similar in appearance to dodders but not taxonomically related, may cause significant losses in cashew yield in Tanzania [34]. Among the stem hemiparasites in the Viscaceae and Loranthaceae families (mistletoes), there are also economically important species, accounting for serious losses in orchards, or forest plants [15]. Recent reports suggest that the problem is persistent and may be expanding [35,36] or have been underestimated previously.

**Figure 1:**
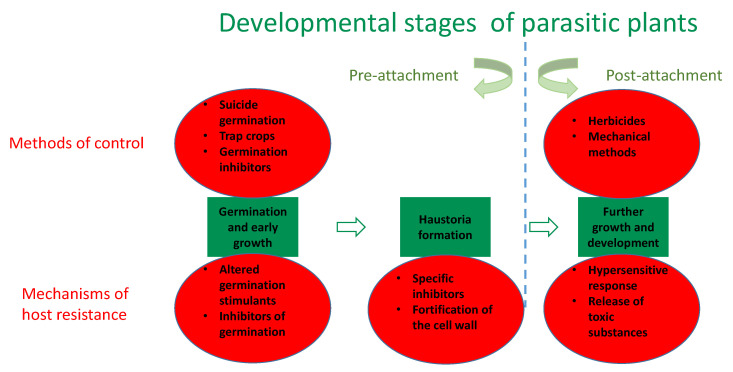
Summary of the most widely applied methods of control and host resistance mechanisms to parasitic plants as dependent on the developmental stages.

Control methods on parasitic plants are often ineffective, due to several important features involved in their strategy for ecological success. First of all, the seeds of both Orobanchaceae and *Cuscuta* spp. are characterized by extreme persistence in soil. They are generally small, thousands are produced by a single plant, and they can stay dormant for decades and germinate only under suitable conditions [37,38,39]. In particular, the seeds of *Striga* spp. and *Orobanche*/*Phelipanche* spp. require the presence of specific germination stimulants, released by potential hosts, called strigolactones in order to emerge [40,41]. Strigolactones are also responsible for the host specificity in the Orobanchaceae family [42,43]. In *Cuscuta* spp., there are no reports on identified germination stimulants. These seed traits ensure continuous irregular germination of the seed bank over an extended time period and the emergence of the parasite only in the presence of a suitable potential host. Various approaches (Figure 1), including suicidal germination induced by synthetic strigolactone analogs applied before sowing of the crop plants [23,44], or trap crops, which induce germination of the parasite, but are resistant to it [45,46], or application of specific seed germination inhibitors [47,48], are often employed with variable success.

The next obstacle to the successful control of parasitic plants is the questionable effectiveness of common pesticides. Being plants on their own, parasitic weeds are subjected to treatment with various herbicides, which affect their respective hosts equally or even more so [14]. Therefore, there is a need for the use of herbicide-resistant crops [29,49]. Finally, complete eradication through mechanical methods is also widely applied, although labor-intensive [50]. Recently, biocontrol methods, including specific pathogens [51,52] or allelopathic interactions [53,54], are also gaining attention as perspective tools in parasitic weed control. A special case here is the identification and selective breeding of resistant host cultivars, which is also the focus of the present review.

## 2. Host Resistance—Molecular Mechanisms

Understanding the molecular mechanisms of host resistance is among the keys to successful cultivar selection. Recently, several review papers have summarized the advance in this research field [55,56,57,58]. The best-established mechanisms are summarized in Figure 1 and Table 2. Host resistance can be divided into pre-attachment and post-attachment [58]. Pre-attachment resistance is further defined as a decrease in parasitic plant seed germination and inhibition of functional haustorial connection, while post-attachment resistance is related to an active immune response against the parasite. The effect of host plants on parasitic plant germination is expected mostly in root parasites, dependent on the specific germination stimulants (e.g., strigolactones) to germinate and localize hosts [40] and not so much in facultative parasites, or stem parasites of the genus *Cuscuta*, for which no such stimulants were proved until now. The differences in the strigolactone synthesis, composition, and release in root exudates were proven as a key element in crop plants’ resistance to *Striga* [59,60,61] and *Orobanche* [27,28]. As far as low strigolactone production may interfere negatively with arbuscular mycorrhizal symbiosis, the selection of cultivars with altered composition instead may be more promising. Furthermore, the exudation of other compounds such as the coumarin scopoletin may contribute to the inhibition of germination, as shown in *O. cumana*-resistant sunflower genotypes [62].

Recognition of the parasitic plant by the host is of key importance for resistance. Indeed, several specific surface receptor proteins were identified in resistant hosts. One such is the CUSCUTA RECEPTOR 1 (CuRe1) in *Solanum lycopersicum*, found to be an essential factor, although not the single factor, responsible for host resistance against *Cuscuta* [63]. Lacking an intracellular kinase domain, CuRe1 is associated with at least two SOBIR1 adaptor kinases to promote its signaling. In sunflower, a putative specific receptor for *O. cumana,* HaOr7 was also identified and further characterized as a leucine-rich repeat receptor-like kinase [64].

Another mechanism is resistance through inhibition of tissue penetration and haustoria formation. This is mainly achieved through fortification of the cell wall in the site of infection through the deposition of callose, suberin, and lignin. Callose deposition as an effective strategy for haustoria inhibition was reported in sunflower resistance to *O. cumana* [65,66] and in Faba bean resistance to *O. crenata* [67]. Lignification of the cell wall was reported in *Trifolium* resistance to *O. minor* [68], in Heinz tomato cultivar resistance to *C. campestris* [69], and in rice resistance to *Striga* [70]. Furthermore, protein cross-linking, mediated by peroxidases, is also a contributing factor to the establishment of a physical barrier for haustoria penetration in pea resistance to *O. crenata* [71] and sunflower resistance to *O. cumana* [65]. Resistant hosts may also express specific inhibitors of haustoria formation, as shown in tomato, expressing specific cysteine protease inhibitor in response to *C. campestris* infection [72].

Successful parasitic plant infection can be disrupted by a hypersensitive response—necrosis of host cells in the site of infection, thus preventing the successful penetration and feeding of the parasite. The hypersensitive response is a widely reported mechanism of resistance to numerous parasitic plants. It was established in sorghum [73] and cowpea [73] genotypes, resistant to *Striga*, as well as in *Cuscuta*-resistant tomato [19,74]. However, such a mechanism was specifically excluded as the basis of resistance, as in resistance to *O. crenata* legumes [75]. Clearly, host resistance to both root and stem parasitic plants shares some common mechanisms, but there are also species-to-species specificities, which should be addressed case by case.

Post-haustorial resistance seems to be the most enigmatic of all. It was extensively reviewed by Yoder and Sholes [56]. Some possibilities include the accumulation of mucilage substance in the host vascular cells, thus blocking the passage of nutrients to the parasite [76], or the expression of specific toxic proteins and/or small interfering RNAs. In the sunflower–*O. cumana* interaction, it was proposed that an increase in phenolic compounds may contribute to post-haustorial resistance [77].

Overall, it should be noted that all these resistance factors are not always all-or-nothing. The response in susceptible host genotypes might be similar to tolerant ones, but not at the level needed for resistance. For example, rice varieties resistant to *Striga hermonthica* do release various strigolactones in root exudates, although in a drastically lower amount than susceptible genotypes [78]. Similarly, strigolactones and scopoletin, found to be involved in sunflower resistance to *O. cumana*, were found to be in higher concentrations in resistant genotypes but were still found in susceptible genotypes [62]. On the other hand, the development of a successful barrier for haustoria formation seems to be a unique feature of resistant genotypes, as shown in *C. campestris*-resistant Heinz tomato cultivar [69], guided by lignification and *O. crenata*-resistant pea cultivars [71], guided by callose accumulation. In all cases, there is a strong genetic basis of resistance [69,78], which can be further used in the selection of parasitic plant-resistant cultivars.

## 3. Cross-Resistance to Parasitic Plants and Other Stresses

It is a well-established fact that plants often display cross-resistance to multiple stresses. This is defined by the common mechanisms of stress response such as universal plant stress hormones, most notably abscisic acid (ABA), salicylic acid (SA), and jasmonic acid (JA) [79], and common antioxidative mechanisms [80], etc. Therefore, tolerance to multiple stresses, whether abiotic, biotic, or a combination of both, is not uncommon in the plant kingdom [81,82]. Although peculiar in its nature due to the close phylogenetic relation between host plants and parasitic plants, plant parasitism is a biotic stress. As such, the response of the host might share similar mechanisms with the response to other biotic stresses, such as insect herbivores and other pathogens [83], as well as abiotic stresses [84].

Therefore, it is tempting to think that the selection of resistant cultivars against one stress factor would offer a multi-resistant cultivar suitable for cultivation under multiple environmental challenges. To name a few examples, the rice cultivar Nipponbare, exemplary for its post-attachment resistance to *Striga hermonthica* [31], was also shown to be resistant against *Schizotetranychus oryzae* (Acari: Tetranychidae) [85], but is salt sensitive [86]. Especially relevant to food securities are several drought-tolerant and *Striga*-resistant maize [87] and cowpea [88] cultivars. In light of climate change, the development of such genotypes with multiple tolerance is extremely important in developing countries. Unfortunately, this is not always the case. For instance, the Faba bean (*Vicia faba* L.) cultivars Misr-1 and Misr-3 showed good resistance to *Orobanche crenata* [89], but high susceptibility to *Fusarium* wilt [90]. In the same crop plant, a certain correlation between salinity tolerance and resistance to *Orobanche* was reported [91]. In certain cases, however, the parasitism may inhibit the response of the host to other biotic stresses, such as herbivores, for example [92]. Several studies suggest that different biotic and abiotic stresses may induce differential expression of responsive genes, as shown in sunflower [93,94]. The *MYB* superfamily showed different expression pattern in response to salinity, drought, and *Orobanche cumana* infection [93]. Unlike it, at least one *bHLH* gene was shown to be similarly affected by drought, cadmium stress, and *Orobanche* [94]. Furthermore, studies in *Vicia faba* suggest the possibility that identification of QTLs, common for *Orobanche crenata* and blight (*Ascochyta fabae*) resistance, is not impossible [95].

At least three common mechanisms could be expected for resistance to both parasitic plants and other stresses. In terms of hormonal control, the SA and JA pathways are good candidates for cross-resistance. They are shown as important regulators of plant response to multiple biotic and abiotic stresses [83,96]. In respect to responses to parasitic plants, they were both shown to be induced in tomato by *C. pentagona* parasitism [97], while in *Trifolium pratense* resistance to *Orobanche minor*, the SA pathway is dominant over the JA pathway [68]. In the plant-to-plant interaction site, the hypersensitive response (HR) of the host, resulting in programmed death of certain cells, was shown as a key mechanism for the prevention of functional haustoria formation in *Sorghum-Striga* [73] and tomato-*Cuscuta* [13] host–parasite pairs. This is also a common response to multiple plant pathogens [98], suggesting that cross-resistance might be expected. Finally, the decrease in plant cell wall permeability through lignification is also a key mechanism of multiple resistance. Along with HR, it is an almost universal mechanism against parasitic plant infestation, as well as one of the best-established resistance mechanisms against multiple pathogens [99]. However, the identification, selection, and development of multi-stress-tolerant crop plant varieties seem to be understudied and more data need to be accumulated in the near future in order to address the increasing need in agricultural practice.

What is missing here is a systemic approach of studying the response of a single host to two different (root and stem) parasitic plants. The existing scientific literature is not abundant in such reports [100] and there seems to be a clear differentiation between Orobanchaceae and *Cuscuta* spp.-affected crop plants. However, the possibility that a single host could be infected by both root and stem parasites simultaneously, and most importantly, that the resistance to one of them would confer resistance to the other, could be of special interest. One such potential model host would be *Solanum lycopersicum*, which has already been discussed as a model for resistance to *Cuscuta*, and is also a common host for several *Orobanche* species. There are numerous studies on tomato resistance to *Orobanche*, and strigolactone-deficiency-related resistance to broomrapes [27] was not correlated with dodder resistance. There are also reports on lignin-based resistance to *Orobanche* [101]. However, it will be intriguing, from an evolutionary aspect, to test whether at least some of the resistance traits to one parasitic plant confer resistance to another.

Another aspect of the multi-stress environment is the often unpredictable interaction between different stresses, or more specifically, the influence of a second stress factor on the host resistance. For example, it was reported, that the ability of an *O. cumana*-resistant genotype to effectively eliminate the infection of the parasite is temperature-dependent and diminishes under lower temperatures [102]. In *C. campestris*, when the host plant is subjected to salinity, the success of the parasite may be significantly affected, either positively or negatively [103]. The *Cuscuta* infection itself may decrease the successful response to herbivores; still, it is not sure whether the vice versa is also true [92]. The parasitic plant-resistant genotypes are supposed to be also highly productive and adapted to the specific environment, but this does not necessarily mean they will perform equally well in a changing environment.

## 4. Selection of Resistant Cultivars

So far, the identification and selection of resistant cultivars of susceptible crop plants appears to be an important strategy by which to decrease the negative impact of parasitic plants on agriculture. In recent years, there have been reports from multiple studies predominantly on crop plants affected by either the root parasites *Striga* spp. and *Orobanche/Phelipanche* spp., or stem parasites *Cuscuta* spp. The most basic approach consists of the large screening of multiple varieties relying on natural resistance. Although a relatively simple approach, it is also laborious, requires large facilities, and there is a high probability of limited success.

Screening for resistance against dodders was conducted in several crop plants with limited success. A single genotype was found to be resistant to *Cuscuta campestris* in a large-scale greenhouse study of 135 accessions of *Vicia sativa* L. and 154 accessions of *Vicia ervilia* (L.) Willd. [26]. In chickpea (*Cicer arietinum* L.), two *C. campestris*-resistant genotypes were established within a collection of 52 genotypes [25]. In carrot resistance to *C. gronovii* some tolerance was observed in four out of ten commercial genotypes [104]. However, this was reported as a lower decrease in biomass of the host, rather than a lack of development of the parasite. Tomato is probably the only crop plant in which *Cuscuta*-resistant cultivars are not an exception [19,69]. Apparently, the success of such an approach is highly limited in dodders, probably because of the broad host range of these parasites, suggesting little to no specialization in respect to the host species [105]. Conversely, there are relatively many cultivars resistant to root parasites, both in sunflower against *O. cumana* [65,77] and *Striga*-resistant cereals [78,88]. This is either because of the more extensive research, the more extensive introduction of resistant varieties in agricultural practice, or because of the higher host specialization of root parasites. However, both *Orobanche* and *Striga* tend to overcome resistance by adapting themselves to resistant hosts in the widely accepted term of gene-to-gene “arms race” [106,107].

A much more sophisticated and effective approach is to map resistance-responsible quantitative trait loci (QTLs) and employ them in marker-assisted selection (MAS). This is neither a new nor a complicated approach, but it gives relatively good results, as reported for *Striga*-resistant sorghum [30], maize [108], and *O. cumana*-resistant sunflower [109]. Furthermore, wild relatives of crop plants are often screened for resistance and resistance-defining genes with putative application in development of parasite-resistant hybrids [110,111,112] and such approaches are getting more and more efficient with the development of high-throughput analytical technologies. Overall, this topic is extremely wide and extensively studied, especially in crop plants susceptible to root parasitic plants. Just like multiple stress tolerance, however, an important issue here is to combine parasitic-resistant traits with other beneficial features, such as high yield, which is addressed in several studies [111,113].

## 5. Host Adaptation to Parasitic Plants—Transgenerational Acquired Resistance?

In respect to stress response, plants are known to respond better when challenged for the second time with the same stressor. For example, seed priming with an abiotic stressor is known to improve germination and performance under salinity [114]. On an individual plant level, exposure to a certain pathogen locally leads to a better response to the same pathogen in distant tissue, or during a next infection. This mechanism is known as systemic acquired resistance (SAR) [115]. Systemic acquired resistance is guided by activation of stress-responsive genes and accumulation of pathogenesis-related proteins. It is among the most important components of plant immunity. In terms of plant–pathogen relationships, the term plant immunity is widespread and often used to describe molecular events resulting from infection. These events cover well-known small interfering RNAs [116], pathogen-associated molecular patterns (PAMP)-induced immunity [117] and SAR [118]. It has been suggested that many of these mechanisms are regulated by epigenetic control [119,120]. There is also evidence for transgenerational resistance to various environmental stressors as a result of epigenetic control [121,122,123]. The progeny of plants subjected to a certain type of stress may show a more effective response to the same stress, which is associated with a massive change in the DNA methylation profile and histone modifications (also termed “stress memory” [124]).

An important question here is whether plants are able to be primed to parasitic plant infections and develop at least partial resistance. It is now well established that genotypic resistance to parasitic plants is genetically determined and inherited [69,78,87]. However, little is known about how these resistant genotypes evolved. In most plants, wild varieties and/or relatives are an established source of genes, involved in parasitic plants resistance as in sunflower [110], maize [125], sorghum [111], and tomato [69]. Therefore, it must be expected that the cultivated varieties have lost this specific trait during the selection process. On the other hand, transgenerational SAR against various pathogens has been reported on numerous occasions [126,127]. If crop plants are continuously challenged by a parasitic plant, this may lead to selective survival of genetic variants with partial resistance, but it could also lead to better performance of the progeny due to epigenetic inheritance. There is still much to be done in this respect. Specific responses to parasitic plants should be studied in the progeny of crop plants continuously subjected to plant parasitism. The pattern of DNA methylation, as well as histone modification, should be compared to transcriptomics analyses in order to establish whether specific genes are differentially regulated under continuous parasitic pressure.

## 6. Conclusions

Parasitic plants, or at least some of them, are among the most damaging pests in agriculture. Accordingly, the application of efficient approaches for control and management is of crucial importance to ensure food security in the light of increasing human population and climate change. Selection of resistant genotypes is an effective, yet not fully exploited, strategy to combat these agricultural pests. Although new data on the genetic and molecular basis of host resistance are accumulating at a significant pace, there are still many knowledge gaps to be filled. Most of the molecular players in host response and resistance to parasitic plants are still unknown. Different host plants display different strategies to combat different parasitic plants, but there are common patterns of response which may be also the key to selecting crop cultivars with multiple stress resistance. There is still also much to be done in terms of understanding the still ongoing co-evolution of parasites and hosts, in order to develop sustainably resistant varieties.

## 7. Future Perspectives

In light of the current knowledge of host resistance to parasitic plants, there are several important questions which must be addressed in future research. First of all, there is a need to define which parasitic plants are established or potential pests and which are important components of biodiversity and natural plant communities. In terms of molecular mechanisms of host resistance, there is still much to be done. However, one underestimated aspect is the cross-resistance/tolerance to multiple stresses, as well as the interaction between them. Finally, the epigenetic regulation of host response and resistance to parasitic plants is a surprisingly understudied area of research. There is also a need to establish a globally accessible collection of parasitic plant genotypes, available to the scientific community, in terms of biodiversity conservation, but most importantly, to facilitate research on host resistance. Especially in *Striga* and *Orobanche* spp., known with their genetic variability, related to significant differences in virulence, it is extremely important to provide the scientific community with the broadest possible parasitic genotypes in order to successfully select for resistant cultivars.

## Figures and Tables

**Table 2:** Overview of the established host plant resistance mechanisms.

Trait	Effect on Parasitic Plants	Affected Parasitic Plants
Altered concentration/composition of strigolactones	Reduced seed germination	Root parasitic plants
Fortification of cell walls by deposition of polymeric substances Local hypersensitive response	Inhibition of tissue penetration Inhibition of tissue penetration/further development	Root and stem parasitic plants Root and stem parasitic plants
Release of specific compounds	Reduced seed germination/inhibition of haustoria formation/inhibition of further development of the parasite	Root and stem parasitic plants, specific to particular species

## Data Availability

Not applicable.
